# Radiolytic Synthesis of Pt-Particle/ABS Catalysts for H_2_O_2_ Decomposition in Contact Lens Cleaning

**DOI:** 10.3390/nano7090235

**Published:** 2017-08-23

**Authors:** Yuji Ohkubo, Tomonori Aoki, Satoshi Seino, Osamu Mori, Issaku Ito, Katsuyoshi Endo, Kazuya Yamamura

**Affiliations:** 1 Graduate School of Engineering, Osaka University, Suita, Osaka 565-0871, Japan; t-aoki@div1.upst.eng.osaka-u.ac.jp (T.A.); seino@mit.eng.osaka-u.ac.jp (S.S.); endo@upst.eng.osaka-u.ac.jp (K.E.); yamamura@upst.eng.osaka-u.ac.jp (K.Y.); 2 Menicon Co., Ltd., Kasugai, Aichi 487-0032, Japan; o-mori@menicon-net.co.jp (O.M.); issaku-ito@menicon-net.co.jp (I.I.)

**Keywords:** nanoparticle, supported catalyst, radical reactions, platinum (Pt), H_2_O_2_ decomposition, contact lens cleaning

## Abstract

A container used in contact lens cleaning requires a Pt plating weight of 1.5 mg for H_2_O_2_ decomposition although Pt is an expensive material. Techniques that decrease the amount of Pt are therefore needed. In this study, Pt nanoparticles instead of Pt plating film were supported on a substrate of acrylonitrile–butadiene–styrene copolymer (ABS). This was achieved by the reduction of Pt ions in an aqueous solution containing the ABS substrate using high-energy electron-beam irradiation. Pt nanoparticles supported on the ABS substrate (Pt-particle/ABS) had a size of 4–10 nm. The amount of Pt required for Pt-particle/ABS was 250 times less than that required for an ABS substrate covered with Pt plating film (Pt-film/ABS). The catalytic activity for H_2_O_2_ decomposition was estimated by measuring the residual H_2_O_2_ concentration after immersing the catalyst for 360 min. The Pt-particle/ABS catalyst had a considerably higher specific catalytic activity for H_2_O_2_ decomposition than the Pt-film/ABS catalyst. In addition, sterilization performance was estimated from the initial rate of H_2_O_2_ decomposition over 60 min. The Pt-particle/ABS catalyst demonstrated a better sterilization performance than the Pt-film/ABS catalyst. The difference between Pt-particle/ABS and Pt-film/ABS was shown to reflect the size of the O_2_ bubbles formed during H_2_O_2_ decomposition.

## 1. Introduction

Three methods are used for cleaning and sterilizing contact lenses: boil cleaning, H_2_O_2_ cleaning, and multipurpose solution (MPS) cleaning. MPS cleaning is a simple method that is used by over 70% of contact lens wearers [1]. However, eye troubles often occur when a lens cleaned with an MPS is not carefully washed. In recent years, the number of wearers using H_2_O_2_ cleaning has gradually increased [1]. When an H_2_O_2_ solution of 35,000 ppm is used for cleaning and sterilizing contact lenses, there is a risk of eyes becoming bloodshot or painful or even of blindness if the H_2_O_2_ solution enters the eyes without being decomposed. H_2_O_2_ decomposition wherein a platinum (Pt) catalyst is used to bring the residual H_2_O_2_ concentration below 100 ppm has therefore been promoted [2]. In the conventional method, a Pt plating film is used as a catalyst and coated on an acrylonitrile–butadiene–styrene copolymer (ABS) container using an electroless plating method. However, Pt is an expensive material. In the Pt plating method, each container requires a loading weight of 1.5 mg. A large quantity of Pt is also used as the catalysts in fuel cells. Techniques that decrease the amount of Pt used for a contact lens cleaning are therefore needed. A further concern is that the reductant used in Pt electroless plating is hazardous to health.

In this study, we therefore attempted to immobilize Pt nanoparticles on an ABS container rather than using a Pt thin film, thereby increasing the specific surface area of Pt. Several methods are used for supporting metal nanoparticles, including the impregnation [3,4,5] and polyol [6,7,8] methods. However, these methods require high temperatures (over 200 °C) and cannot be applied to ABS, which has low heat resistance (less than 100 °C). In a further method, sonolytic synthesis reduces the metal ions in an aqueous solution via irradiation with ultrasound [9,10,11,12]. Although this method has a working temperature of less than 30 °C, the ultrasonic energy is absorbed via the ABS container, leading to nonuniform reduction and deposition of the metal ions. This is a particular problem when the container has a complex three-dimensional shape. Moreover, the productivity of this method is low. Considering these factors, we proposed a technique in which an electron-beam irradiation reduction method (EBIRM) was used to immobilize the Pt nanoparticles on the container. EBIRM is a radiolytic process wherein metal ions in an aqueous solution are reduced via irradiation using a high-energy electron beam. This method has been applied to a range of metal nanoparticles supported on the several particles: γ-Fe_2_O_3_, TiO_2_, and carbon particles [13,14,15]. This method has a number of advantages: no dangerous reductant or catalyst is required, the process requires a low temperature, and the time for electron-beam irradiation is extremely short. This method is particularly suitable in mass production, because a belt conveyor system with a cart can be used for the irradiation process [16].

In this study, we prepared Pt nanoparticles supported on an ABS substrate (Pt-particle/ABS) using the EBIRM. The Pt particle size, Pt loading weight, catalytic activity, sterilization performance, and catalytic durability were investigated.

## 2. Results and Discussion

### 2.1. Particle Size and Pt Loading Weight

**Figure 1:**
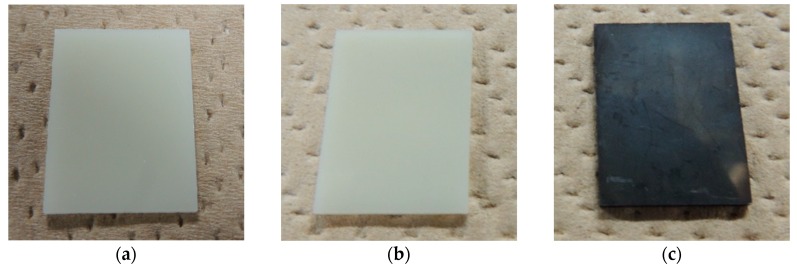
Photographs of the acrylonitrile–butadiene–styrene copolymer (ABS) samples: (**a**) untreated ABS; (**b**) Pt-particle/ABS; and (**c**) Pt-film/ABS.

Some photographs were taken to compare the change of the external appearance. Figure 1 shows the photographs of the untreated ABS, Pt-particle/ABS, and Pt-film/ABS samples. The untreated ABS was yellowish milky white (Figure 1a), whereas the Pt-film/ABS samples appeared blackish (Figure 1c). In contrast, the appearance of the Pt-particle/ABS sample was similar to that of the untreated ABS sample (Figure 1b), to the extent that it was difficult to distinguish between the two based on their external appearance.

**Figure 2:**
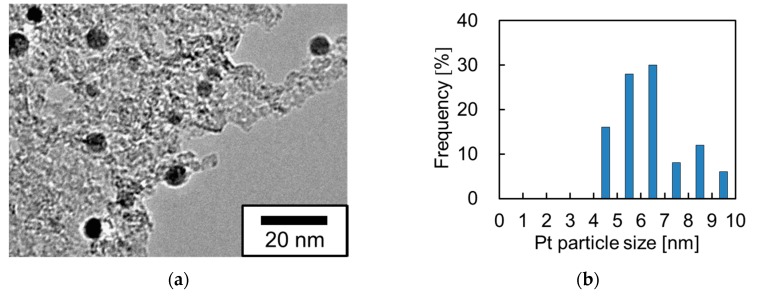
(**a**) TEM image and (**b**) particle-size distribution of Pt-particle/ABS sample synthesized by an electron-beam irradiation reduction method (EBIRM).

To confirm the presence of Pt on Pt-particle/ABS sample, transmission electron microscope (TEM) observation was performed. Figure 2 shows the TEM image and particle-size distribution of the Pt-particle/ABS sample synthesized using the EBIRM. The small black grains with diameters less than 10 nm represent the Pt particles. As shown in Figure 2a, the presence of Pt nanoparticles in the Pt-particle/ABS sample was confirmed and the Pt nanoparticles were shown to be supported by the ABS substrate. Few aggregated Pt particles were observed. The dispersibility of the Pt-particle/ABS was similar to that of Pt-particle/carbon-particle reported in a previous study [15]. The particle sizes of Pt nanoparticles shown in Figure 2b were mainly in the 4–7 nm range, and the average particle size of 6.2 nm was somewhat larger than the particle size of 3.4 nm reported for Pt-particle/carbon-particle by Ohkubo et al. [15]. This difference in average particle sizes is observed due to the different shapes of the support material: substrate and particle and/or the surface properties of the support material: ABS and carbon.

Based on the calibration curve shown in Figure S1, the Pt loading weight of Pt-particle/ABS was only 5.85 μg/substrate. In contrast, the Pt loading weight of Pt-film/ABS was 1500 μg/substrate. The Pt loading weights per unit area of Pt-particle/ABS and Pt-film/ABS were 8.7 and 2240 ng/mm^2^, respectively. Overall, the amount of Pt consumed by Pt-particle/ABS was approximately 250 times less than that of Pt-film/ABS. The Pt specific surface area was also calculated using Equations (1)–(3). The Pt specific surface areas of Pt-film/ABS and Pt-particle/ABS were 0.447 and 45.1 m^2^/g, respectively. The Pt specific surface area of Pt-particle/ABS was 100 times higher than that of Pt-film/ABS.

### 2.2. Catalytic Activity for H_2_O_2_ Decomposition

**Figure 3:**
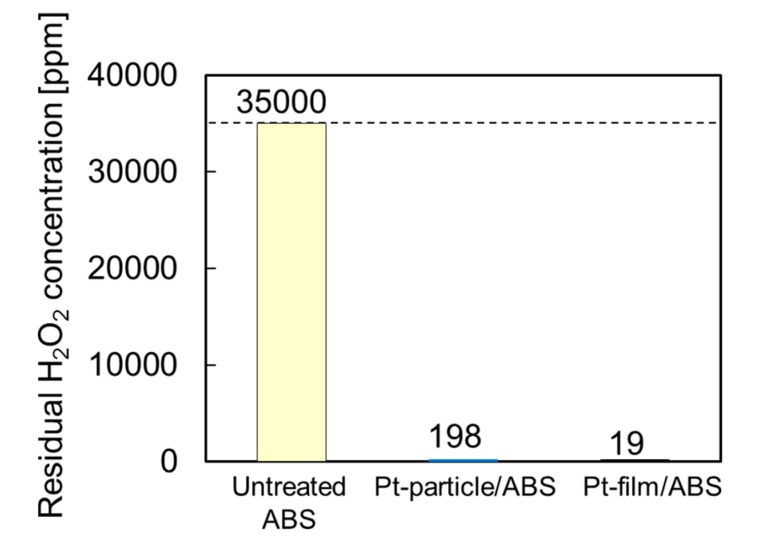
Catalytic activity of the untreated ABS, Pt-particle/ABS, and Pt-film/ABS samples: residual H_2_O_2_ concentration after immersion for 360 min.

To evaluate the catalytic activity for H_2_O_2_ decomposition, residual H_2_O_2_ concentration was measured after the sample was immersed in the H_2_O_2_ solution of 35,000 ppm for 360 min. Figure 3 compares the catalytic activities of the untreated ABS, Pt-particle/ABS, and Pt-film/ABS samples. The untreated ABS sample failed to decompose H_2_O_2_ within 360 min. In contrast, the Pt-particle/ABS sample significantly decreased the residual H_2_O_2_ concentration from 35,000 to 198 ppm. The extremely low Pt loading weight of 8.7 ng/mm^2^ for the Pt-particle/ABS suggests a high level of catalytic activity for H_2_O_2_ decomposition. Although the target value of 100 ppm was not reached, we assumed that it could be achieved by increasing either the area of the ABS substrate and/or Pt loading, because the total Pt surface area of Pt-particle/ABS (266 mm^2^) was less than half that of the Pt-film/ABS (670 mm^2^). Actually, the target value of 100 ppm was successfully reached when the two substrates of Pt-particle/ABS samples were immersed in the H_2_O_2_ solution of 35,000 ppm for 360 min.

### 2.3. Sterilization Performance

**Figure 4:**
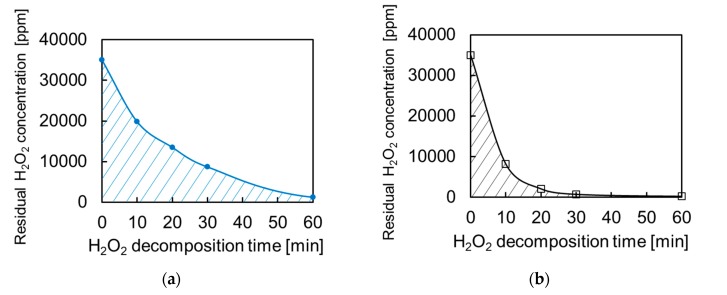
Initial curve of H_2_O_2_ decomposition for the (**a**) Pt-particle/ABS and (**b**) Pt-film/ABS samples.

To evaluate the sterilization performance, the initial H_2_O_2_ decomposition rate was examined. Figure 4 compares the initial curves of H_2_O_2_ decomposition for the Pt-particle/ABS and Pt-film/ABS samples. In both cases, the residual H_2_O_2_ concentration decreased sharply immediately after immersion, after which it decreased gradually. The initial H_2_O_2_ decomposition rate of Pt-particle/ABS was slower than that of Pt-film/ABS. A slower decomposition rate is actually preferable for the sterilization of a contact lens, because excessively fast H_2_O_2_ decomposition leads to incomplete sterilization. An ideal catalyst produces an extremely low initial rate of H_2_O_2_ decomposition; however, it ensures complete neutralization within 360 min. The sterilization performance can be estimated by summing the residual H_2_O_2_ concentration over the initial 60 min. This is shown by the shaded area in Figure 4. It is evident that the Pt-particle/ABS performed better than the Pt-film/ABS sample.

**Figure 5:**
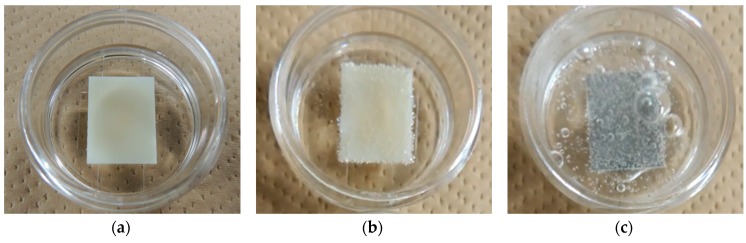
Photographs of the ABS samples: (**a**) untreated ABS; (**b**) Pt-particle/ABS; and (**c**) Pt-film/ABS.

Some photographs were taken in the middle of H_2_O_2_ decomposition for observing the bubble generation and its size. Figure 5 shows the photographs of the untreated ABS, Pt-particle/ABS, and Pt-film/ABS samples soon after the start of H_2_O_2_ decomposition. No bubbles were observed on the untreated ABS sample (Figure 5a). In contrast, many bubbles were observed on both the Pt-particle/ABS and Pt-film/ABS samples (Figure 5b,c, respectively), though with notable differences in the size of the bubbles. The bubbles from the Pt-particle/ABS sample were evidently smaller than those from the Pt-film/ABS sample. Larger bubbles produce greater buoyancy, increasing diffusion through the H_2_O_2_ solution. In brief, the Pt nanoparticles produced smaller bubbles than the Pt film, showing the lower diffusion of the H_2_O_2_ solution and improving the sterilization performance.

### 2.4. Catalytic Durability in H_2_O_2_ Decomposition

**Figure 6:**
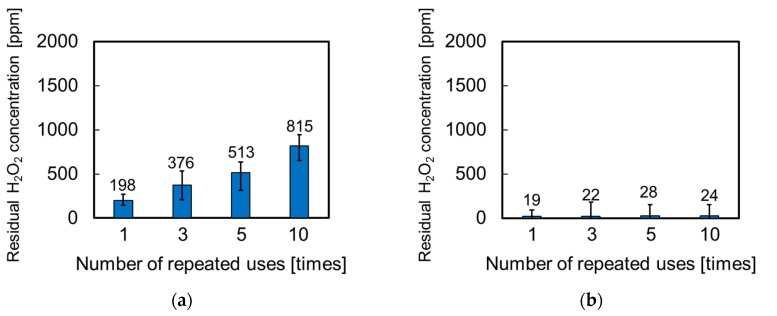
Catalytic durability of the ABS samples: relation between the number of use recycles and residual H_2_O_2_ concentration in (**a**) Pt-particle/ABS and (**b**) Pt-film/ABS.

The relation between the number of repeated uses and residual H_2_O_2_ concentration was examined to evaluate the catalytic durability. Figure 6 shows the catalytic durability of the Pt-particle/ABS and Pt-film/ABS samples. After the Pt-particle/ABS catalysts had been used 10 times, the residual H_2_O_2_ concentration was 815 ppm (Figure 6a). This suggests that Pt remains on the ABS substrate and most of the H_2_O_2_ is decomposed, even after using it 10 times. However, the residual H_2_O_2_ concentration gradually increased as the number of repeated uses increased, suggesting that the catalytic durability was insufficient for use in practical applications. In contrast, the residual H_2_O_2_ concentration of the Pt-film/ABS catalysts was only 24 ppm after using it 10 times (Figure 6b), indicating high catalytic durability. This was due to the persistence of Pt, unless the entire Pt film was removed. There is no need to discuss the turn-over frequency, because there is no chance to poison the Pt surface in the system of H_2_O_2_ decomposition. Actually, a residual H_2_O_2_ concentration of a commercial container covered with a Pt plating film was under 100 ppm after using it 180 times. To estimate the amount of Pt desorption, the Pt loading weights were analyzed via inductively coupled plasma atomic emission spectrometry (ICP-AES) before and after using it 10 times, showing a decrease from 5.85 to 4.76 μg/substrate (from 8.7 to 7.1 ng/mm^2^). For the Pt-particle/ABS catalysts, it is therefore necessary to prevent the desorption of Pt nanoparticles.

**Figure 7:**
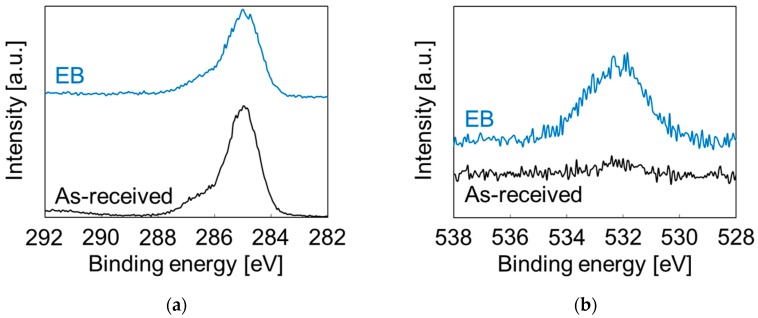
X-ray photoelectron spectroscopy (XPS) spectra of the ABS surface before and after irradiation by an electron beam in water: (**a**) C1s and (**b**) O1s.

To investigate the mechanism by which the Pt nanoparticles were immobilized on the ABS substrate, an untreated ABS substrate was immersed in pure water without Pt precursors and irradiated by an electron beam. Subsequently, the X-ray photoelectron spectroscopy (XPS) and water contact angle (WCA) measurements were performed. Figure 7 shows the C1s- and O1s-XPS spectra of the ABS surface before and after irradiation. The intensity of the peak indexed to C–H and C–C at 285 eV decreased, whereas those of the peaks indexed to C=O at 532 eV and to C–O at 533 eV increased. These results suggested that the ABS surface became oxidized under irradiation by the electron beam. Scission of the polymer chains in acrylic nitrile and/or butadiene rubber and/or polystyrene occur under electron-beam irradiation, which results in generation of carbon radicals. The resulting carbon radicals react with resolved oxygen, active oxygen, hydroxyl radicals, and so on. The WCA also decreased from 78° to 53° upon electron-beam irradiation, suggesting the formation of hydrophilic groups (C–OH or C=O–OH). Our first model assumes the chemical adhesion of Pt nanoparticles through a chemical reaction of these functional groups and/or carbon radicals with Pt ions and/or Pt°. Our second model assumes the unreactive immobilization of Pt nanoparticles through a C–C crosslinking network under electron beam irradiation. In short, Pt nanoparticles were taken into the polymer chain networks. Either one or both models may be correct, and further experiments using a simplex polymer should be conducted to comprehensively clarify the immobilization mechanism. A better understanding of the mechanism will be useful for improving the catalytic durability.

## 3. Materials and Methods 

### 3.1. Synthesis of Pt-Particle/ABS

Pt nanoparticles were immobilized on an ABS substrate using an EBIRM. Elemental processes of the metal ion reduction have been described in previous studies [13,17]. A description of the method for radiolytic synthesis of Pt-particle/ABS follows.

**Figure 8:**
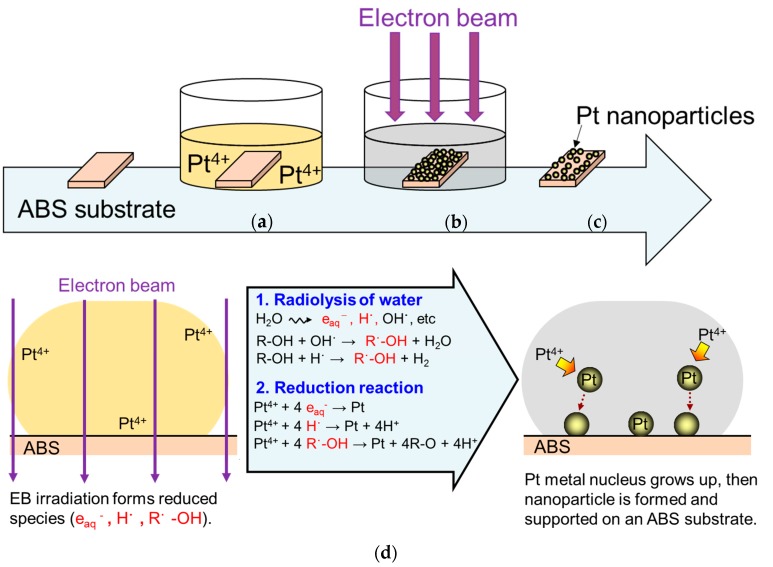
Schematic of the process for preparing a Pt-particle/ABS sample using an EBIRM: (**a**) Immersion of the ABS substrate in Pt precursor solution; (**b**) Irradiation by electron beam; (**c**) Removing it from the solution and drying using an N_2_ gun; and (**d**) Radiolytic synthesis of ABS-supported Pt nanoparticles in detail.

A commercially available 1-mm-thick ABS sheet (2-9229-01, AS-ONE, Osaka, Japan) was cut into pieces of 20 mm × 15 mm × 1 mm for use as the ABS substrate. Prior to use, the ABS substrates were washed with ethanol (99.5%, Kishida Chemical, Osaka, Japan) and pure water for 10 min each using an ultrasonic cleaner (USK-1R, AS-ONE, Osaka, Japan). Subsequently, they were dried using an N_2_ gun (99.99%, Iwatani Fine Gas, Amagasaki, Hyogo, Japan). Hexachloroplatinic acid hexahydrate (H_2_PtCl_6_*·*6H_2_O; 98.5%, Wako Pure Chemical Industries, Osaka, Japan) was used as the Pt precursor, and 2-propanol (IPA; 99.7%, Kishida Chemical, Osaka, Japan) was added as a reduction enhancer. The IPA concentration in the solutions was controlled to be 1 vol %. Aqueous 5-mL solutions containing 4 mM of H_2_PtCl_6_ were prepared in cylindrical polystyrene (PS) containers (diameter = 33 mm and height = 16 mm). The ABS substrate was then immersed in the Pt precursor solutions. To reduce the Pt ions, these precursor solutions were then irradiated with a high-energy electron beam at 4.8 MeV using the Dynamitron accelerator at Japan Electron Beam Irradiation Service Ltd., Osaka, Japan. The surface dose was controlled to be 20 kGy, and each PS container was irradiated for 7 s. After irradiation, the substrates were removed from the solution, washed with pure water using an ultrasonic cleaner for 10 min, and then dried using the N_2_ gun. The entire process is shown in Figure 8. As a comparator, an ABS substrate covered with Pt plating film (Pt-film/ABS) was prepared [18].

### 3.2. Characterization

The dispersibility and particle size of the Pt nanoparticles on the ABS substrate were investigated using a transmission electron microscope (TEM; JEM-2100, 200 kV, JEOL, Akishima, Tokyo, Japan). Specimens were prepared by scratching the surface of the Pt-particle/ABS samples using a V-shaped chisel and dispersing in ethanol using an ultrasonic cleaner. The resulting solution was dropped on a carbon-coated Cu TEM grid (cat. No. 653, Nisshin EM, Shinjyuku, Tokyo, Japan) and dried at 60 °C for over 12 h in a drying machine (AVO-200NS-D, AS-ONE, Osaka, Japan). The particle-size distribution on the Pt-particle/ABS sample was obtained by measuring no fewer than 50 metal particles, and the average particle size was expressed as the geometric mean.

The Pt loading weights of the samples were analyzed using inductively coupled plasma atomic emission spectrometry (ICP-AES; ICPE-9000, Shimadzu, Kyoto, Japan). Pt on the ABS substrate was dissolved using aqua regia with a volume ratio of HCl:HNO_3_ = 3:1. The diluted aqua regia solutions were sprayed into an argon plasma torch through a nebulizer. The amount of Pt in the sample was calculated from the calibration curve using a Pt standard solution (1000 ppm, Wako Pure Chemical Industries, Osaka, Japan), as shown in Figure S1. To derive the calibration curve, the Pt standard solutions were diluted with pure water to 0.01, 0.1, 0.5, 1, 2, 5, and 10 ppm.

The total Pt surface area (*S_total_*) of the Pt-particle/ABS sample was calculated using Equations (1) and (2):

*X* = 4/3 × π × (*R*/2)^3^ × *d* × *a* (1)

*S_total_* = 4 × π × (*R*/2)^2^ × *a*, (2)

where *R* is the diameter of Pt particles (average particle size), *d* is the specific gravity of Pt (21.45 g/cm^3^), *a* is the total number of Pt particles immobilized on an ABS substrate, and *X* is the Pt loading weight. *S_total_* was calculated based on the assumption that all Pt nanoparticles have one-point contact with an ABS substrate or all Pt hemispheres have surface contact with an ABS substrate, and the *S_total_* values in both cases were found to be equal. For calculating *S_total_* of the Pt-film/ABS sample, the Pt film was assumed to not have surface roughness or thickness. The specific surface area (S*_specific_*) of Pt was calculated by dividing *S_total_* by the Pt loading weight (*X*), as shown in Equation (3):

*S_specific_* = *S_total_/X*. (3)

H_2_O_2_ (30%, Kishida Chemical, Osaka, Japan) was diluted to 35,000 ppm with pure water. Five milliliters of the 35,000-ppm solution were added to the PS container, and a Pt-particle/ABS sample was immersed in the solution. The PS container containing the H_2_O_2_ solution and Pt-particle/ABS sample was placed in an incubator (i-CUBE FCI-280, AS-ONE, Osaka, Japan) at 25 °C for 360 min. The Pt catalyst promoted the H_2_O_2_ decomposition, releasing O_2_ gas and decreasing the H_2_O_2_ concentration. The residual H_2_O_2_ concentrations were calculated from adsorption measurements after immersion of the samples for 2, 5, 10, 30, 60, 120, 240, and 360 min. When H_2_O_2_ reacts with Ti(SO_4_)_2_, TiO_2_(H_2_O_2_) is produced, as shown in the following Equation (4):

Ti(SO_4_)_2_ + H_2_O_2_ + 2H_2_O → TiO_2_(H_2_O_2_) + 2H_2_SO_4_ (4)

As shown in Figure S2, the peroxotitanium complex ranged in color from yellow to brownish orange, depending on the residual H_2_O_2_ concentration. Ti(SO_4_)_2_ (30%, Wako Pure Chemical Industries, Osaka, Japan) was diluted to 5% with pure water, and 2 mL of the 5% Ti(SO_4_)_2_ solution was added to the H_2_O_2_ solution. Absorbance measurements of the color change of the solution upon reaction with Ti(SO_4_)_2_ were performed using a deuterium halogen and tungsten lamp (DH-2000, Ocean Optics, Largo, FL, USA), fiber multichannel spectrometer (HR-4000, Ocean Optics), optical fiber (P600-1-UV/VIS, Ocean Optics, Largo, FL, USA), acrylic cuvette (AS-ONE, Osaka, Japan), cuvette holder (CUV-UV, Ocean Optics, Largo, FL, USA), and a cover (CUV-COVER, Ocean Optics, Largo, FL, USA) to eliminate light inclusion. Absorbance at 407 nm was used to calculate the residual H_2_O_2_ concentration from the calibration curve. To derive the calibration curve, the Ti(SO_4_)_2_ solution was diluted with pure water to 10, 30, 60, 100, 300, 500, and 1000 ppm. A representative calibration curve is shown in Figure S3.

The catalytic durability was estimated from the residual H_2_O_2_ concentration after a Pt-particle/ABS sample had been used 1, 3, 5, and 10 times. One cycle comprised three steps: (1) immersion of the Pt-particle/ABS sample in a 35,000-ppm H_2_O_2_ solution at 25 °C for 360 min; (2) removal of the sample from the H_2_O_2_ solution; and (3) drying using the N_2_ gun.

To investigate the effect of the electron-beam irradiation on the surface condition of the ABS substrate, X-ray photoelectron spectroscopy (XPS) and water contact angle (WCA) measurements were performed using Quantum 2000 (Ulvac-Phi, Chigasaki, Kanagawa, Japan) and PG-X (FIBRO System AB, Hägersten, Sweden), respectively. Water droplets with a volume of 3 μL were dropped at seven different points, 1 s after water drop adhesion. The *θ*/2 method was implemented using pocket goniometer software (FIBRO System). The data were averaged, after excluding the lowest and highest values.

## 4. Conclusions

We successfully demonstrated the support of Pt nanoparticles on an ABS substrate using an EBIRM. The amount of Pt required for Pt-particle/ABS was 250 times less than that required for Pt-film/ABS. We also demonstrated that the Pt-particle/ABS catalyst had a considerably higher specific catalytic activity for H_2_O_2_ decomposition than the Pt-film/ABS catalyst. In addition, converting from Pt film to Pt nanoparticles decreased the amount of Pt required for contact lens cleaning while improving sterilization performance. Although the Pt-particle/ABS catalyst retained specific catalytic activity after being used 10 times, the catalytic durability was insufficient for use in practical applications. In our further research, the effect of the surface modification of ABS substrates on catalytic durability will be investigated.

## Supplementary Materials

The following are available online at http://www.mdpi.com/2079-4991/7/9/235/s1. Figure S1: Calibration curve for calculating the amounts of Pt on the Pt-particle/ABS samples: relation between Pt concentration and emission intensity; Figure S2: Photographs of the coloration of peroxotitanium complex: relation between H_2_O_2_ concentration and the color of peroxotitanium complex; and Figure S3: Calibration curve for calculating the H_2_O_2_ concentration: relation between H_2_O_2_ concentration and absorbance at 407 nm.
