# Supplementary material for: Radiolytic Synthesis of Pt-Particle/ABS Catalysts for H2O2 Decomposition in Contact Lens Cleaning
Source: Nanomaterials (Basel). 2017 Aug 23;7(9):235. doi: 10.3390/nano7090235 (PMC5618346; doi:10.3390/nano7090235)

Supplementary Materials

Radiolytic Synthesis of Pt-particle/ABS catalysts for H_2O_2 decomposition in contact lens cleaning

Yuji Ohkubo [1,*], Tomonori Aoki [1], Satoshi Seino [2], Osamu Mori [2], Issaku Ito [2], Katsuyoshi Endo [1] and Kazuya Yamamura [1]

[1] Graduate School of Engineering, Osaka University, 2-1 Yamadaoka, Suita, Osaka 565-0871, Japan [Tel and Fax: +81-6-6879-7294, and E-mail: okubo@upst.eng.osaka-u.ac.jp]

[2] Menicon Co., Ltd, 5-1-10 Takamoridai, Kasugai, Aichi 487-0032, Japan

Contents

- Calibration curve for calculating the amounts of Pt ······························ S1
- Photographs of the coloration of peroxotitanium complex ······················ S2
- Calibration curve for calculating the H_2O_2 concentration ······················· S3

Figure S1 Calibration curve for calculating the amounts of Pt on the Pt-particle/ABS samples: relation between Pt concentration and emission intensity.

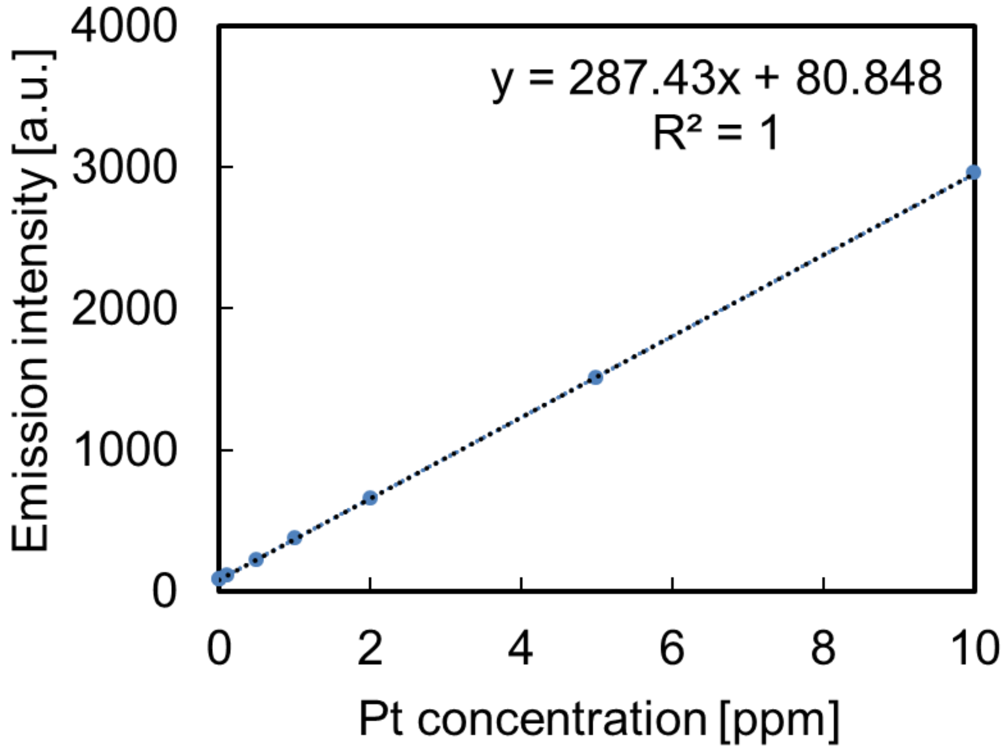

Figure S2 Photographs of the coloration of peroxotitanium complex: relation between H_2O_2 concentration and the color of peroxotitanium complex.

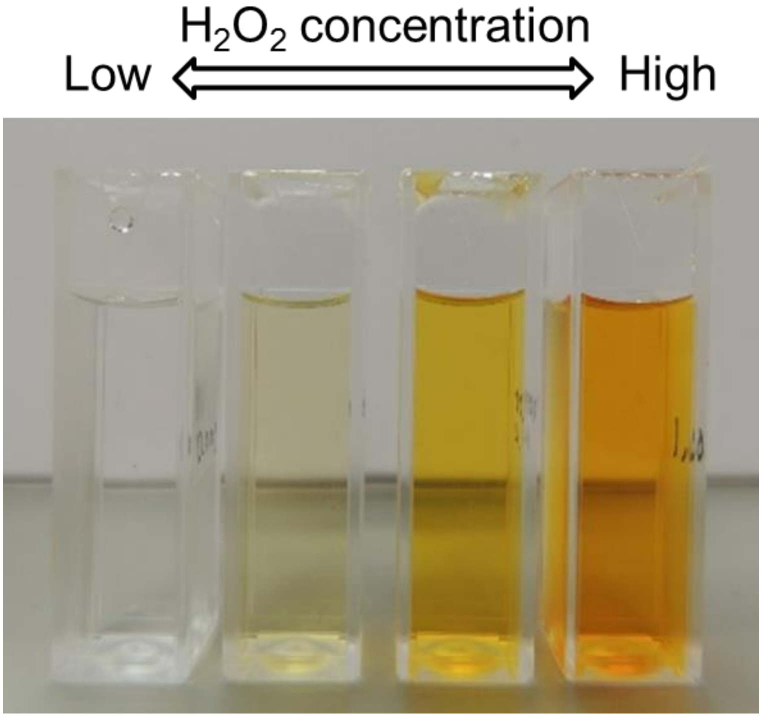

Figure S3 Calibration curve for calculating the H_2O_2 concentration: relation between H_2O_2 concentration and absorbance at 407 nm.

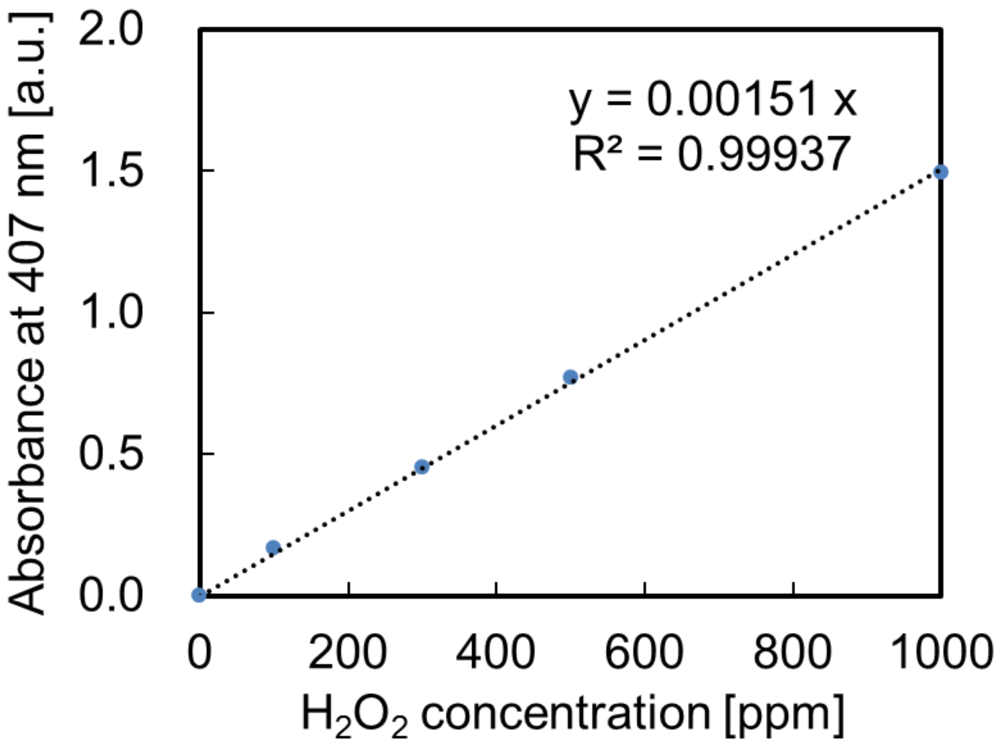

Supplement: Supplementary file 1 [file nanomaterials-07-00235-s001.pdf]
